# Therapeutic Cancer Vaccine and Combinations With Antiangiogenic Therapies and Immune Checkpoint Blockade

**DOI:** 10.3389/fimmu.2019.00467

**Published:** 2019-03-14

**Authors:** Alice Mougel, Magali Terme, Corinne Tanchot

**Affiliations:** ^1^PARCC (Paris-Cardiovascular Research Center), INSERM U970, Paris, France; ^2^UFR Science du Vivant, Université Paris Diderot, Sorbonne Paris Cité, Paris, France; ^3^Faculté de Médecine, Université Paris Descartes, Sorbonne Paris Cité, Paris, France

**Keywords:** cancer vaccines, immunotherapies, combinatorial strategies, antiangiogenic treatments, immune checkpoint blockade

## Abstract

Considering the high importance of immune surveillance and immune escape in the evolution of cancer, the development of immunotherapeutic strategies has become a major field of research in recent decades. The considerable therapeutic breakthrough observed when targeting inhibitory immune checkpoint molecules has highlighted the need to find approaches enabling the induction and proper activation of an immune response against cancer. In this context, therapeutic vaccination, which can induce a specific immune response against tumor antigens, is an important approach to consider. However, this strategy has its advantages and limits. Considering its low clinical efficacy, approaches combining therapeutic cancer vaccine strategies with other immunotherapies or targeted therapies have been emphasized. This review will list different cancer vaccines, with an emphasis on their targets. We highlight the results and limits of vaccine strategies and then describe strategies that combine therapeutic vaccines and antiangiogenic therapies or immune checkpoint blockade. Antiangiogenic therapies and immune checkpoint blockade are of proven clinical efficacy for some indications, but are limited by toxicity and the development of resistance. Their combination with therapeutic vaccines could be a way to improve therapeutic outcome by specifically stimulating the immune system and considering a global approach to tumor microenvironment remodeling.

## Introduction

Therapeutic cancer vaccines are based on specific stimulation of the immune system using tumor antigens to elicit an antitumor response. Nevertheless, therapeutic cancer vaccines are still considered as a strategy that fails to demonstrate clinical benefits. Indeed, when compared to other newly developed immunotherapies such as immune checkpoint blockade (ICB) or CAR T-cell therapies, therapeutic vaccines still show very few outcomes in the establishment of clinical responses in advanced cancer patients.

Numerous improvements have been made in recent decades in therapeutic vaccination protocols that enhance the immune response elicited by the vaccination. FDA approval of the first therapeutic vaccine in 2010, the DC-based vaccine sipuleucel-T (Provenge®) in the treatment of prostate cancer (1), brings to the force possible success for therapeutic vaccination strategies.

In this mini review, we discuss the results and limitations of vaccine approaches tested in clinical trials targeting different tumor antigens. After describing the rationale for combining therapeutic vaccines with either antiangiogenic therapies or ICB, we present examples of combinations in preclinical mouse models and in human clinical trials.

## Therapeutic Cancer Vaccines

### Targeted Antigens

In the context of antitumor vaccine improvement, significant efforts have been focused on the choice of tumor antigen to target. Although numerous cancer vaccine strategies have been studied, targeted antigens remain at the heart of the discussions. They are classified into two main categories.

Tumor-associated antigens (TAAs) are expressed by tumor cells and also by normal cells such as overexpressed antigens (Her2/neu, survivin, MUC-1 …), cancer testis antigens (MAGE-3, NY-ESO-1 …), or differentiation antigens (Mart1, PSA, PAP …). Although TAAs are expressed at a certain level by normal cells, their immunogenicity induces specific T-cell responses (2–4). However, a certain degree of self-tolerance can be applied to TAAs.

In the case of tumor-specific antigens (TSAs), such as oncogenic viral proteins in virally induced cancers or neoantigens generated by non-silent somatic mutations of normal proteins, such central thymic tolerance is bypassed, being regarded as foreign antigens by the immune system. Neoantigen-based vaccine strategies have shown specific anti-tumor immunity in numerous preclinical models and have been tested in early (Phase I) human clinical trials with very promising results (5–10).

### Therapeutic Vaccine Strategies

Different therapeutic vaccine strategies have been developed including whole tumor cell, tumor-cell lysates or gene-modified tumor cells, protein- or peptide-based vaccines, RNA and DNA vaccines, viral vector engineered to express tumor antigen and DC-based vaccines loaded with DNA, RNA or peptides. These strategies are further detailed in specialized reviews (10–15).

Many preclinical and clinical trials using these different strategies have been performed and we will focus here on those that have reached phase II/III clinical trials (16, 17). Some examples are provided below and in Table 1 (18–29).

**Table 1:** Some examples of phase II/III clinical trials testing therapeutic cancer vaccines.

**Vaccine**	**Cancer type**	**Trial phase**	**Trial outcomes**	**ClinicalTrials.gov Identifier and References**
**DC-BASED VACCINE**				
Peptide-pulsed DCs	Advanced melanoma	Phase III	DC vaccination was ineffective compared to chemotherapy	Schadendorf et al. (18)
**VACCINE USING VIRAL VECTOR**				
TG4010: MVA expressing MUC-1 and IL-2	mRCC	Phase II	Induction of an immunological response against MUC-1 has been observed and safety has been established. No clinical benefit following vaccination	Oudard et al. (19)
TG4010: MVA expressing MUC-1 and IL-2	NSCLC	Phase II (TIME clinical trial)	Patients showing a MUC-1-specific response (*n* = 16) had an improved clinical outcome with a median overall survival (OS) of 32.1 vs. 12.7 months in non-responders (*n* = 6)	NCT01383148 Tosch et al. (20)
TroVax: MVA expressing fetal oncogene 5T4 (MVA-5T4)	Renal cancer	Phase III	No clinical benefit	NCT00397345 Amato et al. (21)
PROSTVAC (or PSA-TRICOM): MVA expressing PSA	mCRPC (metastatic castration-resistant prostate cancer)	Phase II	44% reduction in death rate and an 8.5 month improvement in median OS	NCT00078585 Kantoff et al. (22)
**TUMOR CELL-BASED VACCINE**				
GVAX	Metastatic hormone-refractory prostate cancer	Phase I/II	Dose escalation study. The immunotherapy was well-tolerated. The median survival time was 35.0 months in the high-dose group, 20.0 months in the mid-dose group and 23.1 months in the low-dose group	NCT00140348 Higano et al. (23)
Allogenic GM-CSF secreting tumor	Pancreatic adenocarcinoma	Phase II	Vaccine combined with chemoradiation is safe. Median disease-free survival was 17.3 months and median OS was 24.8 months, which compares favorably with published data	NCT00084383 Lutz et al. (24)
**PROTEIN/PEPTIDE-BASED VACCINE**				
recMAGE-A3 protein + AS15 immunostimulant	Completely resected stage IB, II, and IIIA MAGEA3+ NSCLC	Phase III	Vaccination failed to increase the disease-free survival of surgically resected NSCLC patients	NCT00480025 Vansteenkiste et al. (25)
Rindopepimut with GM-CSF plus temozolomide	Newly diagnosed EGFRvIII+ glioma patients	Phase III	Rindopepimut did not increase survival in newly diagnosed glioblastoma patients	NCT01480479 Weller et al. (26)
IMA901: 10 different synthetic tumor-associated peptide	mRCC	Phase II	Vaccination was safe and well-tolerated. Median OS was 23.5 months for patients pretreated with cyclophosphamide	NCT00523159 Walter et al. (27)
Tecemotide (L-BLP25): MUC-1-derived lipopeptide	NSCLC (stage III)	Phase III	No significant difference in OS.	NCT00409188 Butts et al. (28)
Multiepitope vaccine composed of tyrosinase, gp100 and MART-1 peptides	High-risk resected melanoma	Phase III	No RFS or OS improvement in vaccinated patients	NCT01989572 Lawson et al. (29)

Cancer vaccine strategies based on autologous dendritic cells (DCs) pulsed with tumor antigens have been widely studied (30, 31). For example, a phase III clinical trial testing peptide-pulsed DCs as a first-line treatment in advanced melanoma patients has been performed, but DC vaccination was ineffective compared to chemotherapy (18). A randomized phase II/III clinical trial is currently ongoing in glioblastoma patients to test a DC-based vaccine (NCT03548571). Many parameters such as DC-based vaccine administration, maintenance of DC viability and maturation as well as standardization of *ex vivo* generation can be limiting (32–34).

Improvement of vaccine platforms has also led to the use of viral vectors with modified viruses engineered to express both targeted antigens and immunomodulatory molecules. In this context, modified vaccinia virus of the Ankara strain (MVA) has been studied. A vaccine containing MVA expressing tumor antigen MUC-1 and immunostimulatory cytokine IL-2 (TG4010 vaccine) was tested in a phase II clinical trial in patients with metastatic renal cell carcinoma (mRCC) (19). Induction of an immunological response against MUC-1 was observed and safety established. Nevertheless, the trial showed no clinical benefit following vaccination. In the TIME clinical trial (phase II), TG4010 was tested in NSCLC patients with or without chemotherapy. Patients who showed a MUC-1-specific response (*n* = 16) had an improved clinical outcome with a median overall survival (OS) of 32.1 months vs. 12.7 months in non-responders (*n* = 6) (20).

Other vaccines have also emerged using this modified virus strategy, such as TroVax, an MVA expressing fetal oncogene 5T4 (MVA-5T4) studied in a phase III clinical trial in the treatment of renal cancer, though no clinical benefit was found (21). PROSTVAC (or PSA-TRICOM), a poxviral-based prostate-specific antigen (PSA) vaccine has been assessed in a phase II clinical trial in the treatment of metastatic castration-resistant prostate cancer (mCRPC) and was associated with a 44% reduction in the death rate and an 8.5 months improvement in median OS (22).

Thus, therapeutic vaccine strategies take many forms and importance is now also given to vaccine administration routes to enhance efficacy (35) as well as work done on vehicle delivery and adjuvants (12, 15, 36–38).

### Therapeutic Vaccines in the Era of Combination Strategies

However, even though cancer vaccines have been greatly improved, overall they still fail to provide any clinical benefit as monotherapy in patients with advanced cancers. During cancer progression, tumors develop several mechanisms of immune escape such as tumor angiogenesis, recruitment of immunosuppressive cells, and over-expression of inhibitory molecules, all leading to an ineffective antitumor immune response (39, 40).

Due to their lack of migration and/or progressive exhaustion, tumor-specific T cells generated by vaccination do not act effectively against the tumor. Combination with strategies that counteract such immune escape mechanisms is therefore essential.

Therapeutic vaccine approaches have a major place in the arsenal of weapons developed to fight cancer and in many published studies have been combined with radio- or chemotherapy, with promising results, as reviewed elsewhere (41, 42).

In this mini review, we will concentrate on combinations of therapeutic vaccines and antiangiogenic treatments (AATs) or ICB, highlighting their synergistic potential and providing an update of preclinical and clinical results.

## Therapeutic Cancer Vaccines and Antiangiogenic Treatments Combinations

### Rationale for Vaccine Combination With AATs

During carcinogenesis, tumors will increase the expression of pro-angiogenic molecules such as vascular endothelial growth factor (VEGF), which is involved in tumor angiogenesis. This process fosters access to nutrient and oxygen supplies for the tumor and allows proliferation and metastatic dissemination even though vasculature development is abnormal (43). On the other hand, the aberrant tumor vasculature actively suppresses anti-tumor responses by providing a physical barrier to T cell infiltration and limits therapeutic drug efficacy due to poor delivery to the tumor (40). Tumor angiogenesis also contributes to immune escape with its immunosuppressive roles (44–47). For example, VEGF is involved in inhibition of DC maturation, development of tumor-associated macrophages, increase in regulatory T cells (Tregs), accumulation of myeloid-derived suppressor cells (MDSCs), and expression of inhibitory checkpoints such as PD-1 on CD8^+^ T cells (48).

Within this framework, numerous antiangiogenic molecules have been developed to repress tumor angiogenesis. AATs target many components of the tumor microenvironment (endothelial cells, tumor cells, DC, MDSC, Tregs….) resulting in a shift of cytokine and chemokine production favoring antitumor activities. These therapies lead to transient vascular normalization while dampening immunosuppression (40, 47, 49, 50). In this review, we will focus on drugs targeting the VEGF/VEGF receptor (VEGFR) pathway, such as bevacizumab (an anti-VEGFA antibody), tyrosine kinase inhibitors such as sunitinib, sorafenib or axitinib, which target VEFGR but also other pathways, and anti-VEGFR antibodies. These antiangiogenic drugs are currently the most used in the clinical setting. However, the benefits provided by these treatments are still limited and acquired resistance can appear (51, 52).

Considering the extensive tumor microenvironment remodeling induced by AATs, a combination of such strategies with therapeutic vaccines could help to enhance the immune response against tumors. By improving in quantity and quality the infiltration of T lymphocytes activated by the vaccine and by decreasing immunosuppression, AATs might act in synergy with therapeutic cancer vaccines.

### Preclinical Studies Combining AATs And Vaccines

Several preclinical studies have sought to define the best timing of administration of therapeutic vaccines and AATs for an optimal synergy of action. These studies have yielded conflicting results.

In a first study, sunitinib was combined with a DC-based vaccine expressing IL-12 and pulsed with OVA-peptide (DC-IL12-OVA) in a B16-OVA tumor model. This combination improved therapeutic efficacy, increased type-1 antitumor T-cell recruitment in the tumor microenvironment and decreased immunosuppressive cells (MDSCs, Tregs) (53). Similar results in terms of efficacy were obtained using the tyrosine kinase inhibitor axitinib instead of sunitinib (54).

Bose et al. showed that the best therapeutic efficacy was achieved when sunitinib was administered alongside vaccine priming or boosting, whereas starting sunitinib treatment after vaccination did not lead to optimal efficacy (53).

Farsaci et al. studied the combination of sunitinib with a modified virus-based vaccine using rMVA containing transgenes for co-stimulatory molecules (B7-1, ICAM-1, and LFA-3) as well as for the carcinoembryogenic antigen (CEA) administered to CEA-transgenic mice bearing MC38-CEA tumors (55). Sunitinib was administered for 4 weeks followed by 2 weeks without treatment (comparable to schedules in cancer patients). In this context, vaccine was administered before, alongside or after the start of sunitinib treatment. Only situations where sunitinib preceded vaccination were associated with increased therapeutic efficacy when compared to sunitinib alone. The authors concluded that synergy between sunitinib and therapeutic vaccination happened after sunitinib preconditioned the immune system.

Finally, a study combining sunitinib with a protein-based vaccine using recombinant α-lactalbumin in a 4T1 mammary tumor-bearing mouse model highlighted the fact that sunitinib inhibited the priming phase of the active immunization protocol by reducing the number of CD11b^+^ CD11c^+^ antigen-presenting cells in draining lymph nodes and spleen when sunitinib is administered alongside vaccination (56).

Besides the importance of the timing of administration, the dose of antiangiogenic treatment is a key factor for vessel normalization in the tumor (47, 49). In a preclinical model of breast cancer, only low doses of anti-VEGFR2 antibody (DC101) enabled vascular normalization, and a combination of low-dose DC101 with irradiated tumor cell vaccine enhanced tumor control compared to vaccine alone (57).

Although there seems to be no consensus regarding treatment scheduling, some patterns do emerge. The nature of the vaccine strategy should be taken into account, as should the antiangiogenic drug properties, considering the large spectrum of immune responses considered.

### Clinical Trials Combining AATs And Vaccines

Based on positive results in preclinical studies, the combination of AATs and therapeutic vaccines in human subjects is currently being assessed. In a phase II clinical study involving newly diagnosed intermediate and low-risk mRCC patients, sunitinib was combined with a personalized DC-based vaccine AGS-003 consisting of monocyte-derived DCs transfected with total autologous tumor RNAs and CD40L RNA. Vaccine administration was started at the beginning of the second cycle of sunitinib. Combination treatment induced immunological responses and prolonged survival (58). These encouraging results led to a phase III clinical trial, but AGS-003 failed to improve OS (NCT01582672).

The same lack of clinical response has been reported in a phase II clinical trial combining the multipeptide IMA901 with sunitinib in the treatment of mRCC (59).

Although progress has been made in the understanding of the impact of AATs, combinatorial approaches with cancer vaccines have failed to demonstrate clinical efficacy. To improve clinical outcomes, before considering vaccination we need a better understanding of immunological remodeling induced by antiangiogenic therapies and an assessment of the immunological stage of patients already on antiangiogenic drugs in the context of standard of care treatment. On the other hand, as mentioned above, attention should be paid to the treatment schedule and the doses of AAT. Clinical trials are usually not design to address this issue. To date, the combination of vaccines and antiangiogenic therapies has been less studied than other combinations, although numerous clinical trials are ongoing (some are listed in Table 2A) and hopefully will provide new insights into how such strategies might optimize synergistic effects.

**Table 2:** Some examples of ongoing clinical trials combining cancer vaccines and antiangiogenic therapies **(A)** or immune checkpoint blockade **(B)**.

**(A) Ongoing clinical studies combining cancer vaccines and antiangiogenic therapies**					
**Vaccine**	**Antiangiogenic therapy**	**Other therapy/adjuvants**	**Cancer type**	**Trial phase and status**	**ClinicalTrials.gov Identifier**
Peptide vaccine (EGFRvIII, EphA2, Her2/neu peptide)	Bevacizumab		Glioblastoma	Phase II: active, not recruiting	NCT02754362
HSPPC-96 (personalized peptide-based vaccine)	Bevacizumab		GBM	Phase II: active, not recruiting	NCT01814813
Intuvax (allogenic cell-based therapy)	Sunitinib	poly-ICLC +/– KLH	mRCC	Phase II: active, not recruiting	NCT02432846
PF-06755990 (vaccine)	Sunitinib	Tremelimumab	Prostate cancer	Phase I: recruiting	NCT02616185
**(B) Ongoing clinical studies combining cancer vaccines and immune checkpoint blockade**					
**Vaccine**	**Immune checkpoint blockade**	**Other therapy /Adjuvants**	**Cancer type**	**Trial phase and status**	**ClinicalTrials.gov Identifier**
NeoVax (neoantigen peptide)	Pembrolizumab	Radiation Therapy	Glioblastoma	Phase I: active, not recruiting	NCT02287528
Peptide vaccine	Pembrolizumab		Pancreatic ductal adenocarcinoma or colorectal adenocarcinoma	Phase I: recruiting	NCT02600949
DPX-Survivac (encapsulated peptide)	Pembrolizumab	Cyclophosphamide	Advanced ovarian, primary peritoneal or fallopian tube cancer	Phase II: recruiting	NCT03029403
pTVG-HP (DNA vaccine encoding PAP antigen)	Nivolumab	GM-CSF	Non-metastatic, PSA-recurrent prostate cancer	Phase II: recruiting	NCT03600350
GVAX (GM-CSF-secreting tumor cells)	Nivolumab	Cyclophosphamide	Pancreatic cancer	Phase I/II: recruiting	NCT02451982
PROSTVAC (poxviral vector expressing PSA)	Nivolumab		Pancreatic cancer	Phase I/IIa: recruiting	NCT02933255
PROSTVAC (poxviral vector expressing PSA)	Ipilimumab		Pancreatic cancer	Phase II: recruiting	NCT02506114
GVAX (GM-CSF-secreting tumor cells)	Nivolumab, ipilimumab	CRS-207, Cyclophosphamide	Pancreatic cancer	Phase II: recruiting	NCT03190265
Dendritic cell-based p53 vaccine	Nivolumab, ipilimumab		Relapsed small cell lung cancer	Phase II: recruiting	NCT03406715
Neoantigen DNA vaccine	Durvalumab		Triple negative breast cancer	Phase I: recruiting	NCT03199040
CDX-1401 vaccine (DEC-205/NY-ESO-1 fusion protein)	Atezolizumab	Guadecitabine	Recurrent ovarian cancer	Phase II: recruiting	NCT03206047

*Bevacizumab, anti-VEGF-A mAb; sunitinib, tyrosine kinase inhibitor; pembrolizumab and nivolumab, anti-PD-1 mAbs; ipilimumab and tremelimumab, anti-CTLA-4 mAbs; durvalumab and atezolizumab, anti-PD-L1 mAbs*.

## Therapeutic Cancer Vaccines and Immune Checkpoint Blockade Combinations

### Rationale for Vaccine Combination With ICB

As described before, the acquisition of immune checkpoint molecules by effector T cells in the tumor microenvironment render them progressively exhausted and unable to kill tumor cells. This has led in recent years to the development of ICB, which is now used in the treatment of many types of cancer.

Monoclonal antibodies (mAbs) directed against co-inhibitory molecules involved in T cell exhaustion or Treg cell function such as CTLA-4 (cytotoxic T-lymphocyte-associated antigen 4) and the PD-1/PD-L1 axes (programmed cell death 1/programmed death-ligand 1) have revolutionized the treatment of an increasing number of cancers, including melanoma, lung cancer, renal cancer, bladder cancer, and Hodgkin's lymphoma (60).

Although success stories such as anti-CTLA-4 and anti-PD-1 mAbs have emerged, these strategies seem to work as monotherapies in a restricted number of patients and some limitations have emerged, with the development of acquired resistance (61, 62). It is important to highlight that poorly immunogenic tumors (also referred to as “cold” tumors), like pancreatic and prostate cancers, are not sensitive to checkpoint blockade (63). Accordingly, the rationale of combining ICB with therapies that increase the number of infiltrating tumor-specific T cells, such as vaccination, is often underlined (63, 64).

### Preclinical Studies Combining ICB And Vaccines

Anti-CTLA-4 mAbs in combination with cancer vaccines have been tested in some preclinical studies. For instance, a GM-CSF-producing tumor cell vaccine combined with CTLA-4 blockade has important synergistic effects in reducing tumor size and increasing the antitumor immune response in a melanoma model (65) and in a prostate cancer model (66).

Wada et al. have demonstrated the importance of timing with the combination of anti-CTLA-4 mAbs and GM-CSF gene-transfected tumor cell (GVAX) vaccine in the prostate cancer model Pro-TRAMP (67), by showing that anti-CTLA-4 mAbs should be administered after vaccination to produce additive effects. They hypothesized that delayed CTLA-4 blockade could avoid compensatory expansion of the Treg compartment, which might affect generation of an effective antitumor response.

To improve treatment of pancreatic ductal adenocarcinoma in which checkpoint inhibitor monotherapies seem to be ineffective, Soares et al. have shown that combining anti-PD-1 with GVAX vaccine improved survival and enhanced T-cell activity in mice (68).

Similar conclusions have been reached with another non-immunogenic tumor model. The combination of DC tumor lysate-based vaccine with PD-1 mAbs resulted in long-term survival in mice bearing large established glioma tumors, while neither treatment alone improved survival (69).

In addition, a preclinical study revealed that PD-1 checkpoint inhibition combined with an adenoviral-based vaccine targeting HPV-E6/E7 protein in the context of E6^+^/E7^+^ tumor-bearing mice resulted in a more effective antitumor response (70). The authors also showed that treatment with vaccine alone upregulated the expression of several inhibitory immune checkpoint molecules, including PD-1 and LAG-3 on CD8^+^ tumor-infiltrating lymphocytes. Another study reported increased PD-1 expression on CD8^+^ tumor-infiltrating lymphocytes after vaccination and significantly enhanced tumor growth inhibition when vaccine was combined with anti-PD-L1 (71). These results strengthen the rationale for vaccine and ICB combination and suggest that multiple checkpoint inhibition could help enhance the synergy of action with vaccine strategies.

Following this idea, Duraiswamy et al. showed that dual blockade of PD-1 and CTLA-4 combined with GVAX vaccine resulted in the rejection of CT26 colorectal tumor in 100% of mice and of ID8-VEGF ovarian carcinoma in 75% (72). Murine studies therefore support the concept that anti-CTLA-4 and anti-PD-1 mAbs increase the frequency of activated T cells and the effector T cell to Treg ratio in vaccinated tumors. While studies have focused on the scheduling of anti-CTLA-4 mAbs with vaccination, this issue has been less studied for the combination of anti-PD-1 mAbs and vaccine.

### Clinical Trials Combining ICB And Vaccines

Following preclinical studies, clinical trials have tested the efficacy of such combinatorial approaches in cancer patients.

In melanoma patients, peptide vaccines have been tested in combination with ipilimumab (anti-CTLA-4), but were not associated with improved outcomes compared to ipilimumab alone (73–75). Nevertheless, some of these peptide vaccines have not shown great immunogenicity when tested alone in preclinical trials.

But combinatorial trials have also yielded promising results. In a phase Ib study, ipilimumab was tested in combination with GVAX vaccine in patients with pancreatic adenocarcinoma. The combination of the vaccine with anti-CTLA-4 therapy improved OS compared to ipilimumab alone (76). In another study, the authors showed that antibody-mediated CTLA-4 blockade increases tumor immunity in some patients who were previously vaccinated with GVAX (77).

In a phase I clinical trial, MART-1 peptide-pulsed DCs combined with tremelimumab (anti-CTLA-4) resulted in objective and durable tumor responses in melanoma patients (78). Of 16 patients, 4 had an objective response, 2 had a partial response, and 2 a complete response. TriMixDC-MEL, autologous monocyte-derived DCs electroporated with synthetic mRNA, combined with ipilimumab in advanced melanoma has shown an encouraging rate (38%) of highly durable tumor responses in a phase II trial, including 8 complete responses and 7 partial responses (79).

Viral vector-based vaccines combined with ICB have also shown great promise in human studies. The PROSTVAC vaccine combined with ipilimumab in treatment of mCRPC in a phase I dose-escalation trial proved safe, but clinical outcome has not been studied (80) and a randomized phase II trial is currently recruiting (NCT02506114). Aside from CTLA-4 blockade, PROSTVAC efficacy is also being assessed when combined with nivolumab (anti-PD-1) (phase I/II recruiting) (NCT02933255).

Clinical studies of a combination of anti-PD-1 antibodies and vaccines are still limited, but some early trials show encouraging results.

In a phase I study, patients with advanced solid cancers received p53MVA vaccine combined with pembrolizumab (anti-PD-1) (81). Clinical responses were observed in 3/11 patients, in whom disease remained stable for 30, 32, and 49 weeks, associated for two of them with an increased frequency and persistence of p53-reactive CD8^+^ T cells.

In a single-arm, phase II clinical trial, 24 patients with incurable HPV-16–positive cancer were vaccinated with ISA 101, a synthetic long-peptide vaccine composed of overlapping HPV E6 and E7 peptides in combination with nivolumab (82). The overall response rate of 33% and median OS of 17.5 months are promising compared to the 16–22% overall response rate and median survival of **~**9 months with PD-1 inhibitors alone in similar patients (63).

Two phase I studies have shown that nivolumab in combination with peptide vaccines targeting differentiation antigens is safe and produces an immunological response in melanoma patients (83, 84).

Although clinical data on the combination of anti-CTLA-4 or anti-PD-1 mAbs and vaccines are still limited, some phase I or II clinical trials are ongoing (85). Table 2B lists ongoing clinical trials combining those mAbs but also involving PD-L1 blockade with therapeutic vaccines with or without more conventional therapies (radiotherapy, chemotherapy).

Regarding the complexity of assessing real clinical improvement in such combinatorial trials, an effort should be made in terms of trial design to determine whether the efficacy of vaccine-induced immune responses is improved when combined with immune checkpoint inhibition. It will also be necessary to evaluate immune mechanisms involved in the response to treatment in patients. Those parameters will then allow better scheduling for combination therapy, depending on the nature of the therapeutic vaccine and inhibitory molecules used.

## Conclusions and Perspectives

Although therapeutic cancer vaccines have been associated with past failures, the era of combinatorial strategies in the treatment of cancer prompts their reconsideration. Strategies have been optimized and immunologic enhancement due to vaccines is now accepted. The overwhelming immunosuppressive tumor microenvironment that reduces the clinical efficacy of vaccines can now be modified by different approaches. Combinations of cancer vaccines and antiangiogenic therapies or ICB have emerged and shown promising results. To date, very impressive results for those combinations described in mice have not yet been recapitulated in humans. However, studies in mice have mainly used sub-cutaneous tumor grafts growing rapidly and representing an early stage of the disease. Conversely, clinical trials mainly concern patients with advanced cancers, i.e., at a late phase of the disease when immunosuppressive mechanisms are induced. Consequently, we currently lack clinical data showing any breakthrough, a better understanding of the tumor microenvironment will allow us to consider new combinations. Questions remain concerning the timing of treatments, adjuvants, immunization routes, optimal immunogenic vaccines, and tumor remodeling. There is also a need to set up clinical trials in patients at early disease stages. Combinations including newly developed ICB or costimulatory pathways as well as other antiangiogenic strategies such as vaccines directly targeting angiogenic compounds could also bring new hope and lead to clinical success. Finally, in the near future, multiple therapies involving distinct but complementary aspects of antitumor responses may be considered as the combination of vaccines, antiangiogenic therapies and ICB.

## Author Contributions

All authors listed have made a substantial, direct and intellectual contribution to the work, and approved it for publication.

### Conflict of Interest Statement

The authors declare that the research was conducted in the absence of any commercial or financial relationships that could be construed as a potential conflict of interest.
